# Effectiveness and Safety of an Outpatient Program for Percutaneous Left Atrial Appendage Occlusion

**DOI:** 10.3390/jcm12216728

**Published:** 2023-10-24

**Authors:** Fabián Blanco-Fernández, Pablo J. Antúnez-Muiños, Jean C. Núñez-García, Sergio López-Tejero, Gilles J. Barreira-de Sousa, Mónica García-Monsalvo, Milena Antúnez-Ballesteros, Andrew Maree, David González-Calle, Javier Rodríguez-Collado, Manuel Barreiro-Pérez, Elena Díaz-Peláez, María C. Pérez del Villar-Moro, Pedro L. Sánchez-Fernández, Ignacio Cruz-González

**Affiliations:** 1 Department of Cardiology, Hospital Universitario de Salamanca, 37007 Salamanca, Spainguillesdesousa@gmail.com (G.J.B.-d.S.);; 2 Instituto de Investigación Biomédica de Salamanca, 37007 Salamanca, Spain; 3 Centro de Ivestigación Biomédica en Red—Enfermedades Cardiovasculares (CIBER-CV), 37007 Salamanca, Spain; 4 Hospital Universitario de Burgos, 09006 Burgos, Spain; 5 St. James’s Hospital, D08 NHY1 Dublin, Ireland; 6 Hospital Álvaro Cunqueiro, 36213 Vigo, Spain

**Keywords:** stroke prevention, atrial fibrillation, oral anticoagulation, left atrial appendage occlusion, LAAO, LAAC, interventional cardiology

## Abstract

Background: Left atrial appendage occlusion (LAAO) is a safe and effective alternative to oral anticoagulation for thromboprophylaxis in patients with nonvalvular atrial fibrillation. Technological development in devices and imaging techniques, as well as accumulated experience, have increased procedural success rates and decreased complications. Same-day discharge protocols have been proposed in the field of structural heart disease, but this approach has not been studied in detail for the LAAO procedure. Aim: The aim of this study is to assess the safety and efficacy of an outpatient program for LAAO when compared to the conventional treatment approach. Methods: We present a retrospective, non-randomized single-center study of 262 consecutive patients undergoing LAAO. Patients were divided into two groups, the first (*n* = 131) followed a conventional protocol (CP), and the second (*n* = 131) an outpatient protocol (OP). The primary composite endpoint comprised MACCE (death, stroke, and bleeding), cardiac tamponade, vascular complication, or attendance in the emergency department after hospital discharge at 30 days. Results: The overall success rate was 99.6%, with a periprocedural complication rate of 2.29%. With regards to the CP versus OP group, there were no differences between incidences of the primary composite endpoint (6.1% PC vs. 3.0% PA, *p* = 0.24), or after an analysis, with propensity score matching. No differences were observed in the individual endpoints. There was a decrease in hospital length of stay in the same-day discharge group (*p* < 0.01). Conclusions: A same-day discharge LAAO program is safe, effective, and feasible when compared to the conventional strategy. Moreover, it reduces hospital length of stay, which might have clinical and economic benefits.

## 1. Introduction

Atrial fibrillation (AF) is the most common sustained arrhythmia in the general population [1], and it significantly increases the risk of thromboembolic events. Different factors have been analyzed as potential predictors for these thromboembolic events and different risk scores established to guide anticoagulation use (CHADS2 and CHA2DS2-VASc) [2]. Oral anticoagulation with vitamin K antagonists and, more recently, with direct oral anticoagulants have been included in clinical practice guidelines and are considered first-line treatment to reduce thromboembolic events in patients with AF [3].

In over 90% of the cases of patients with nonvalvular AF, the thrombi form in the left atrial appendage [4]. Therefore, left atrial appendage occlusion (LAAO) has emerged as a safe and effective therapeutic alternative to vitamin K antagonists [5,6,7,8,9] and direct oral anticoagulants [10]. The number of these procedures is constantly increasing, as well as operator experience [11,12,13]. Furthermore, technological advances in devices and imaging techniques have significantly increased procedural success rate and decreased rates of complications [12,13]. In this context, outpatient same-day discharge procedures have been proposed to reduce the length of hospital stays and thereby the related costs and complications. To our knowledge, an outpatient strategy for LAAO has only been assessed in three single-center series and, indirectly, in a multi-center study [14,15,16,17], but has never been compared to the conventional strategy directly or after adjusting for other factors.

The objective of this study is to assess the safety and efficacy of an outpatient program for LAAO when compared to the conventional treatment approach.

## 2. Methods

This is a retrospective, single-center study that included 262 consecutive patients who underwent LAAO from March 2017 to August 2021. The first group included patients who followed the standard protocol with admission for at least 24 h (conventional protocol, *n* = 131). The second group included cases starting from October 2019, when an outpatient strategy was established by default, based on the early same-day discharge of patients (outpatient protocol; *n* = 131). Both strategies are detailed below.

### 2.1. Conventional Protocol (CP)

The patients were admitted the morning of the procedure to the cardiology day-case hospital. After the procedure, the patients returned to the day-case hospital for monitoring. After 4–5 h, they were transferred to the ward if no complications were observed after clinical reassessment and an echocardiogram. If no incidents appeared, the patients were discharged 24 h after the procedure and after a further transthoracic echocardiogram.

### 2.2. Outpatient Protocol (OP)

The patients were admitted in the morning of the procedure to the cardiology day-case hospital. After the procedure, if no acute complications were observed, they were transferred to the day-case hospital again and monitored. After 4–5 h, an echocardiogram was conducted, and the attending cardiologists carried out a clinical assessment. The patients were discharged after confirming that there were no complications.

### 2.3. Procedure

Prior to the intervention, a cardiac imaging test was conducted (either transesophageal echocardiogram or CT scan) to rule out the presence of thrombi in the left atrial appendage. A morphological study of the atrial appendage and its dimensions was also carried out in order to choose the device and its size. Finally, the interatrial septum was analyzed to plan the transseptal puncture.

The technique for left atrial appendage closure has already been described [17,18]. In our center, since March 2017, the cases are guided by default with a pediatric micro echocardiogram probe [19]. In these cases, conscious sedation was administered for a more comfortable procedure for the patients. Alternatively, in cases in which the transesophageal echocardiogram with a 3D probe was necessary, the procedure was conducted with general anesthesia, orotracheal intubation, and mechanical ventilation.

The femoral vein was accessed with vascular ultrasound guidance in all cases. After the procedure, the vascular access was closed with a figure-of-eight suture, which was removed 4 h later, and the patient was monitored to rule out bleeding.

Technical success was defined as adequate deployment and implant of the device without complication [20]. Procedural success was defined as when the implant is achieved without significant residual shunt (<5 mm) [20]. Device used was at the operator’s discretion.

### 2.4. Main Objectives

The primary composite endpoint to assess safety and effectiveness was MACCE (death, stroke, and bleeding), together with vascular complications and the need to receive emergency health care within the first 7 days after hospital discharge, analyzed at 30 days. The secondary endpoints were the technical success and procedure success, average length of stay in both groups, and complications during the procedure and the hospital stay.

### 2.5. Statistical Analysis

An intention-to-treat analysis was conducted. Continuous variables are expressed as mean ± standard deviation or median and interquartile range, and normality was assessed with the Shapiro–Wilk test. Variables with a normal distribution were compared by Student’s *t*-test. Non-normally distributed variables were analyzed with the Mann–Whitney U test. Qualitative variables are expressed as percentages and compared by the chi-squared test.

The chi-squared test was also used to compare the incidence of the composite event and the individual events. Also, the Cox regression model was used to assess the impact of the outpatient program versus the conventional protocol on survival rates, or adverse events during or after the procedure.

Considering the potential differences between baseline characteristics in both groups, the patients were matched based on their propensity score. Matching used the nearest neighbor method, with a 1:1 ratio and a caliper width of 0.2, and it included all variables with significant differences between both groups and/or variables with clear clinical relevance. After the adjustment, 76 pairs of patients were identified and compared by the same methods. Results were expressed as hazard ratios and analyzed with Kaplan–Meier survival curves for 30 days.

A bilateral *p*-value < 0.05 was considered statistically significant.

Statistical analysis was performed using Stata 15.1.

## 3. Results

### 3.1. Basal Characteristics

Table 1 summarizes the basal characteristics of both groups and the matched groups using propensity scores. The study of the hemorrhagic and ischemic risk scales (CHA2DS2, CHA2DS2-VASc, and HAS-BLED) shows a significant increase in ischemic risk (CHADS2, 3.3 vs. 2.8, *p* < 0.01) and in hemorrhagic risk (HAS-BLED, 3.9 vs. 3.1, *p* < 0.01) in the CP group.

### 3.2. Procedure

The main characteristics of the procedure are described in Table 2.

Conventional protocol: The rate of procedural success (defined as occlusion without residual leaks >5 mm in the echocardiogram during the procedure) was 99.2% (130/131) of the cases in this group. The procedure was accompanied by a second intervention in two cases (one revascularization of the circumflex artery, being an elective procedure not related to a complication of the procedure, and the closure of an aortic prosthetic leak). More than one device was used in seven cases (5%), six of which required two devices and one which required three devices. There was only one case of a significant complication during the procedure, which comprised an episode of cardiac tamponade that required emergent pericardiocentesis.

Outpatient protocol: The rate of procedural success was 100%, without significant residual leakage on the echocardiogram. A combined procedure took place in three cases (one case of percutaneous coronary revascularization, one case of patent foramen ovale closure and one case of pulmonary vein isolation). More than one occlusion device was required in five cases.

### 3.3. Hospital Discharge and Stay

In the conventional protocol group, the mean length of stay was 24 h. Of the patients, 83.2% (109/131) were discharged 24 h after the procedure. The remaining patients (22/131) had a mean stay of 87 h with a maximum stay of 288 h for one patient.

On the other hand, the mean length of stay in the outpatient protocol group was 8.7 h. Only 8 patients (6%) required hospital admission (in three cases because they required a 3D probe and general anesthesia), with a mean stay of 32 h and a maximum stay of 48 h.

Hospital length of stay was significantly shorter in the OP group (*p* < 0.01).

### 3.4. Complications during Admission

In-hospital complications occurred in a total of 11 patients (4.19%) and were considered significant and related to the procedure in six cases (2.29%). Nine of these events occurred in the CP group and included five cases of vascular complication (two arteriovenous fistulas, one pseudoaneurysm, and two inguinal hematomas) which did not require surgical correction, one case of periprocedural stroke, one cardiac tamponade, one case of bleeding unrelated to the procedure which required transfusion, and one case of death caused by global respiratory insufficiency in a patient with COPD which became exacerbated during admission.

### 3.5. Need for Health Assistance after Discharge

Admissions to the emergency department within the first 7 days after the procedure were analyzed. In the CP group, six patients were re-admitted due to an emergency, but in only one case was the cause related to the procedure (access site bleeding that required a simple suture for hemostasis). On the other hand, the OP group had five emergency admissions, and only one of them was directly related to the LAAO procedure. It was also a mild femoral hemorrhage that was resolved with a simple suture.

### 3.6. Safety and Effectiveness

**Figure 1:**
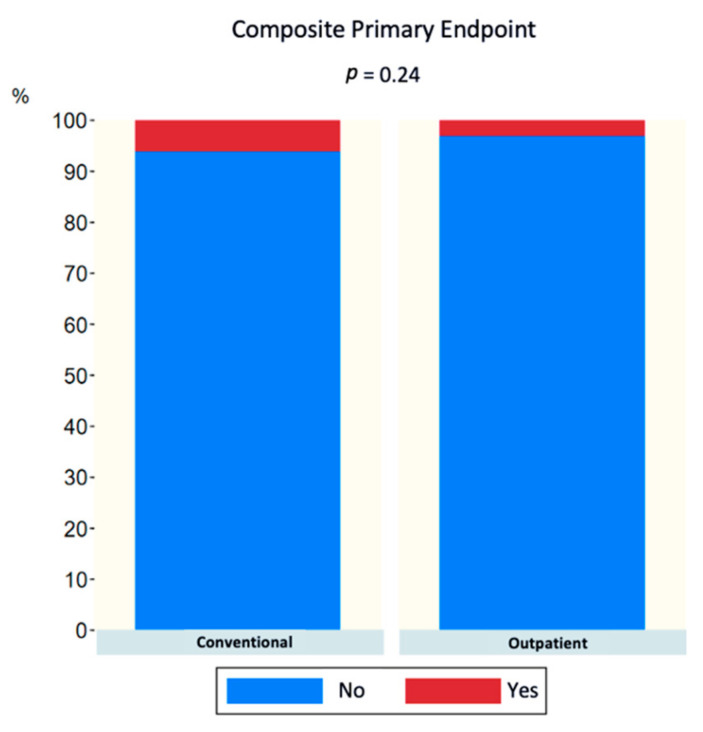
Bar graph of composite primary endpoint (MACCE + cardiac tamponade + vascular complication or attendance in the emergency department after hospital discharge) at 30 days.

**Figure 2:**
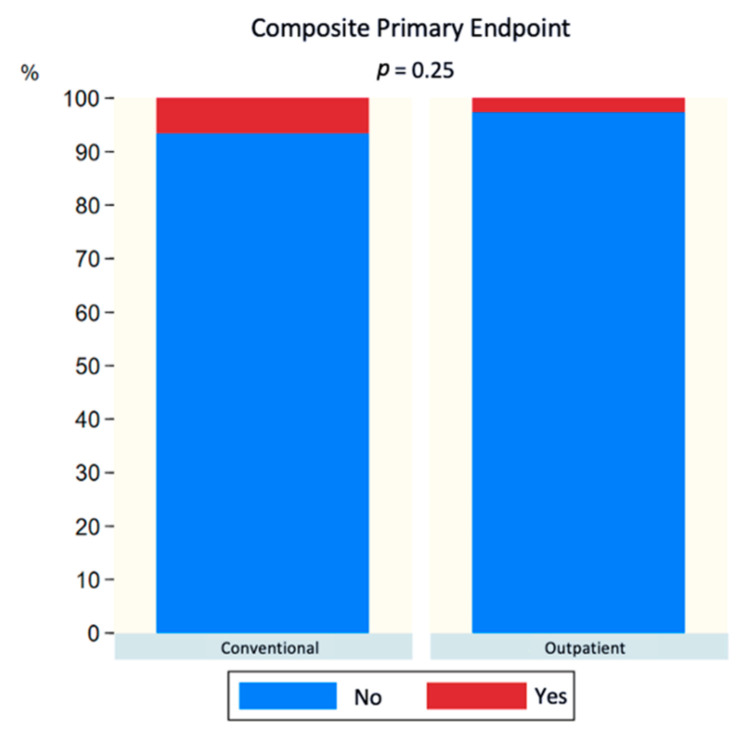
Bar graph of composite primary endpoint (MACCE + cardiac tamponade + vascular complications or attendance in the emergency department after hospital discharge) at 30 days after propensity score matching.

**Figure 3:**
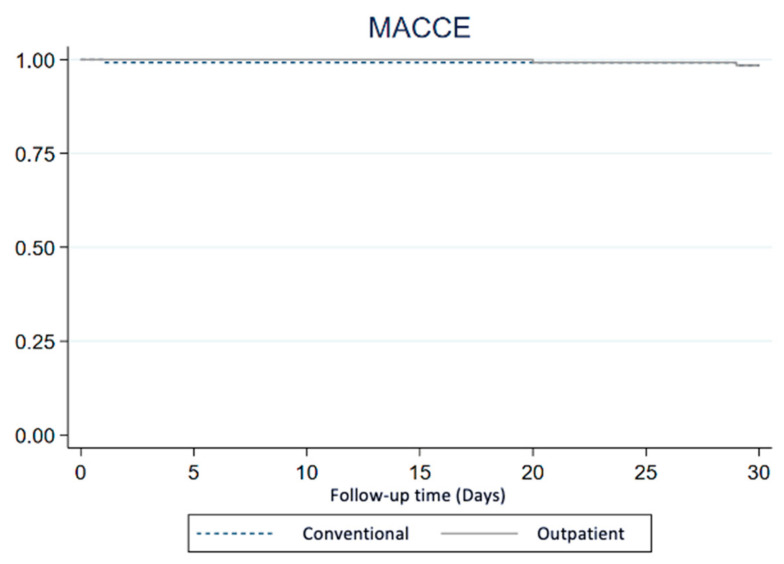
MACCE-free survival analysis (including death, stroke, and bleeding) at 30 days.

**Figure 4:**
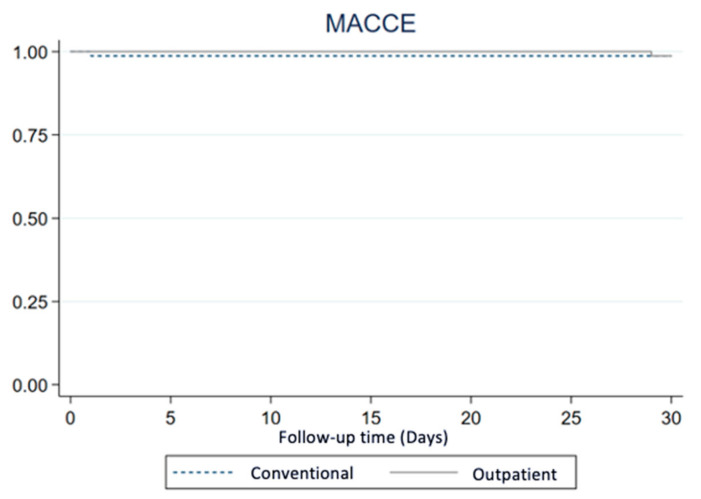
MACCE-free survival analysis (including death, stroke, and bleeding) at 30 days after propensity score matching.

In the analysis of the composite primary endpoint (MACCE, vascular complications, and need for emergency care within the first 7 days after the procedure), no statistically significant differences were observed after 30 days (6.1% in CP vs. 3.0% in OP, *p* = 0.24) (Figure 1). The same analysis was conducted for the pairs established with propensity score matching (matched for age, sex, AHT, previous heart failure, CHADS2, HAS-BLED, and method of anesthesia), and no statistically significant differences were observed between both groups (6.6% in CP vs. 2.6% in OP, *p* = 0.25) (Figure 2). In addition, each component of the composite event was analyzed individually, and no statistically significant differences were observed, as can be seen in Table 3. MACCE-free survival analysis (including death, stroke, and bleeding) after 30 days was performed, and no statistically significant differences were observed between both groups (HR 0.99 (0.13–7.03) *p* = 0.992) (Figure 3) or after propensity score matching (HR 0.99 (0.06–15.88) *p* = 0.996) (Figure 4).

Main Figure: Non-randomized single-center study with two groups, conventional protocol *n* = 131 and outpatient protocol *n* = 131 (76 in each group after matching). No differences were observed in the composite primary endpoint.

Table 3 shows the isolated analysis of the components of the primary composite endpoint, finding no statistically significant differences in death, stroke, bleeding, and MACCE, nor after stratification by propensity score. An important aspect is the need for emergency care in the first week (which would be avoided with admission for 24 h), in which no differences were observed either. Finally, similar results were found for complications directly related to the procedure, such as vascular complications or relevant pericardial effusion.

## 4. Discussion

This study compares a program of left atrial appendage occlusion following an outpatient protocol in which patients are discharged on the day of the intervention with the conventional protocol that includes admission for at least 24 h. Our results show that there are no differences in the short-term safety and effectiveness between strategies, and that the OP approach achieves a significant decrease in hospital stay, with corresponding potential clinical and socioeconomic benefits.

Left atrial appendage occlusion is an effective alternative to oral anticoagulation with antivitamin K drugs. The PROTECT study confirmed that LAAO was non-inferior to oral anticoagulation with a composite endpoint of stroke, systemic embolism, and cardiovascular death [5,6,9]. Given the initial high rate of complications, the PREVAIL study was conducted, and it revealed a lower number of complications and confirmed the safety of the procedure [7]. After a 5-year follow-up of both randomized studies, LAAO met superiority criteria for a composite endpoint of hemorrhagic stroke, cardiovascular death, and death from all causes [9]. Afterwards, the PRAGUE 17 trial proved that LAAO was not inferior to direct-acting anticoagulants for a combined endpoint of thromboembolic events, cardiovascular death, clinically significant hemorrhage, and device-related complications [10]. There are multiple records that confirm the safety and effectiveness of this procedure in patients with contraindications to oral anticoagulation [11,21,22,23,24,25].

Our study presents the results of LAAO procedures from March 2017 to August 2021 in a single large-volume center with significant operator experience. The global characteristics of our population are similar to those in other available studies and registries of LAAO [11,21,22,23], and the average age was slightly higher than in recent registries [11,21]. Thrombotic risk in our cohort was high, with an average CHA2DS2-VASc score of 4.46, which is similar to other registries, such as EWOLUTION [21]. Hemorrhagic risk was also high, with an average HAS-BLED score of 3.48, which is slightly higher than rates in the EWOLUTION trial and similar to the Belgian registry that included 457 LAAO [22]. In our series, technical success of device implantation was achieved in 99.6% of patients and procedure success without significant leak (≥5 mm) in 99.2% of the CP cases and 100% of the OP group. This was consistent with the results observed in recent registries such as EWOLUTION [21] and the American registry (99.8%) [11].

Outpatient protocols have been implemented safely and effectively in several procedures in the field of interventionist cardiology, such as the diagnosis and treatment of coronary disease [26], supraventricular arrhythmia ablation [27,28], or simple structural procedures such as patent foramen ovale closure. More complex procedures, such as percutaneous implantation of transcatheter aortic valve prostheses, are already implementing a minimalist approach with early discharge [29,30].

Advances in imaging techniques [31,32,33,34] and improvement in device design, as well as accumulated experience, have made it possible to implement a less invasive approach in LAAO, without reducing the success rates of the procedure and with fewer complications. To our knowledge, few studies have evaluated an outpatient approach with LAAO. Williams T et al. [14] carried out a single-center retrospective study without a comparison group with a cohort of 117 patients and confirmed a low rate of periprocedural complications and a high percentage of same-day discharge without incident. E-Xin Tan et al. [15] conducted a retrospective analysis of 211 patients and did not observe differences between patients who were discharged on the same day and patients who were hospitalized. However, their study excluded patients with an unsuccessful occlusion, patients who suffered complications during the procedure and patients who were admitted for clinical reasons prior to the occlusion. In addition, early discharge was decided based on the preferences of the patients or their relatives, and these inclusion and exclusion criteria may bias the results. Palma-Dallan et al. [16] compared 23 patients with early discharge to 119 patients who followed a conventional protocol, but all the patients with early discharge underwent procedures with intracardiac echocardiography, whereas those in the conventional protocol received transesophageal echocardiography. Finally, an analysis of the American readmissions database [17] compared the outpatient protocol with the conventional strategy, and no differences were observed regarding the number of readmissions. However, that study did not analyze procedural success, complications, or emergency treatment.

This is the first study that compares two retrospective cohorts of patients who underwent LAAO and presents a direct and adjusted comparison of results that includes effectiveness and safety between a conventional admission strategy and an outpatient approach. The analysis did not reveal statistically significant differences between groups with regards to the main composite endpoint at 30 days (MACCE + vascular complications + attention in the emergency services within the first 7 days after discharge). In order to prevent bias caused by differences between the groups, an analysis with propensity score matching for clinically relevant variables was performed and, again, no significant differences were observed regarding the composite event, the individual events, or the success rate. Regarding the effectiveness, no differences were observed in the percentage of technical success or the success of the procedure, which was high in both groups. With regard to safety, a low number of complications were observed and did not differ significantly between groups. Reassuringly, the outpatient protocol did not result in higher emergency attendance within the first seven days. Finally, our study shows a significant reduction in length of hospital stay in the OP group, with the potential clinical and socioeconomic benefits this represents.

### Limitations

The main limitation in this study is the non-randomized nature of the analysis, which is a retrospective study of two cohorts of consecutive patients, which could result in selection bias. However, no differences were observed in the analysis after propensity score matching. Furthermore, this is a single-center study based on a single team of image operators/specialists with significant LAAO procedural experience, which may make it difficult to extrapolate the results to other departments.

## 5. Conclusions

An outpatient protocol for LAAO with same-day discharge is safe and effective and significantly reduces the duration of hospital stay.

## 6. Impact on Daily Practice

Safety or efficacy did not differ between the outpatient strategy and the conventional approach.

Early discharge in OP LAAO significantly reduces hospital stay, with potential clinical and socioeconomic benefits.

## Figures and Tables

**Table 1:** Basal characteristics.

Basal Characteristics	Conventional Protocol (*n* = 131)	Outpatient Protocol (*n* = 131)	*p*	Conventional Protocol (Matched *n* = 76)	Outpatient Protocol (Matched *n* = 76)	*p*
Age	78.7 ± 9.2	78.6 ± 8.9	0.94	78.6 ± 10.1	77.8 ± 8.5	0.58
Gender (male)	82.0 (62.6%)	81.0 (61.8%)	0.90	49.0 (64.5%)	49.0 (64.5%)	0.99
BMI	25.0 (24.0–28.0)	27.0 (24.0–30.0)	0.10	25.0 (24.0–28.0)	28.0 (24.0–30.0)	0.06
Hypertension	117 (89.3%)	104 (79.4%)	0.03	65 (85.5%)	66 (86.8%)	0.81
Dyslipidemia	70 (53.4%)	71 (54.2%)	0.90	42 (55.3%)	37 (48.7%)	0.42
Diabetes	52 (39.7%)	41 (31.3%)	0.16	29 (38.2%)	28 (36.8%)	0.87
Smoke	18 (13.9%)	10 (7.6%)	0.10	11 (14.5%)	4 (5.3%)	0.05
Previous Stroke	56 (42.8%)	46 (35.1%)	0.20	33 (43.5%)	33 (43.4%)	0.99
Previous systemic embolism	30 (23.1%)	3 (2.3%)	<0.01	15 (19.7%)	2 (2.6%)	<0.01
Hemodialysis	9 (6.9%)	6 (3.8%)	0.27	4 (5.3%)	3 (3.9%)	0.70
Previous bleeding	112 (85.5%)	101 (77.1%)	0.08	63 (82.9%)	59 (77.6%)	0.41
Ischemic cardiopathy	25 (19.2%)	23 (17.6%)	0.73	13 (17.1%)	11 (14.5%)	0.66
LVEF < 40%	9 (6.9%)	11 (8.4%)	0.64	2 (2.6%)	9 (11.8%)	0.03
Heart failure	47 (35.9%)	30 (22.9%)	0.02	19 (25.0%)	19 (25.0%)	0.99
Cancer	33 (25.2%)	34 (26.2%)	0.86	23 (30.3%)	20 (26.7%)	0.62
CHADS2	3.3 (3.1–3.5)	2.8 (2.6–3.0)	<0.01	3.1 (2.8–3.3)	3.2 (2.9–3.4)	0.72
CHADS2VASC	4.6 (4.4–4.8)	4.3 (4.1–4.5)	0.07	4.4 (4.0–4.7)	4.7 (4.4–5.0)	0.11
HASBLED	3.9 (3.7–4.0)	3.1 (3.0–3.2)	<0.01	3.4 (3.3–3.6)	3.5 (3.3–3.6)	0.91
GFR	58.0 (39.0–76.0)	60.0 (40.0–79.0)	0.52	58.0 (40.5–76.5)	62.5 (39.5–78.5)	0.83
Paroxysmal AF	46 (35.7%)	49 (38.6%)	0.63	28 (36.8%)	25 (32.9%)	0.70
Previous IC bleeding	30 (25.2%)	20 (15.3%)	0.047	19 (25.0%)	14 (18.4%)	0.33
Previous GI bleeding	66 (50.4%)	70 (53.4%)	0.62	34 (44.7%)	37 (48.7%)	0.63
Basal Hb (g/dL)	11.9 (10.2–13.4)	11.9 (10.3–13.8)	0.54	12.2 (10.2–13.6)	11.7 (10.1–13.8)	0.79
Platelets (×10^3^/µL)	188.0 (145.0–232.0)	194.5 (162.5–248.5)	0.10	198.5 (158.0–253.5)	200.0 (162.0–252.0)	0.62

AF: Atrial fibrillation. LVEF: Left ventricular ejection fraction. GFR: Glomerular filtration rate. GI: Gastrointestinal. IC: Intracranial. BMI: Body mass index.

**Table 2:** Main characteristics of the procedure.

Main Characteristics of the Procedure	Conventional Protocol (*n* = 131)	Outpatient Protocol (*n* = 131)	*p*	Conventional Protocol (Matched *n* = 76)	Outpatient Protocol (Matched *n* = 76)	*p*
General anesthesia	25 (19.1%)	5 (3.8%)	<0.01	3 (3.9%)	5 (6.6%)	0.47
Micro TE probe	103 (78.6%)	110 (84.0%)	0.27	68 (89.5%)	62 (81.6%)	0.17
ICE	4 (3.1%)	9 (6.9%)	0.16	4 (5.3%)	4 (5.3%)	0.99
Procedural success	130 (99,2%)	131 (100%)	0.99	76 (100.0%)	76 (100.0%)	0.99
Contrast (mL)	115.0 (89.0–160.0)	86.0 (70.0–108.0)	<0.01	114.5 (85.0–159.0)	83.5 (66.0–102.5)	<0.01

TE: Transesophageal echocardiography. ICE: Intracardiac echocardiogram.

**Table 3:** Follow-up characteristics.

Follow-Up Characteristics	Conventional Protocol (*n* = 131)	Outpatient Protocol (*n* = 131)	*p*	Conventional Protocol (Matched *n* = 76)	Outpatient Protocol (Matched *n* = 76)	*p*
Death (30 days)	2 (1.5%)	2 (1.5%)	0.99	1 (1.3%)	1 (1.3%)	0.99
Stroke (30 days)	1 (0.8%)	0 (0.0%)	0.32	0 (0.0%)	0 (0.0%)	---
Bleeding (BARC ≥ 3)	2 (1.5%)	1 (0.8)	0.56	0 (0.0%)	1 (1.3%)	0.32
MACCE (30 days)	3 (2.3%)	2 (1.5%)	0.65	1 (1.3%)	1 (1.3%)	0.99
Attendance in the emergency department < 7 d related to the procedure	1 (0.8%)	1 (0.8%)	0.99	1 (1.3%)	0 (0.0%)	0.32
Vascular complications	3 (2.3%)	0 (0%)	0.08	2 (2.6%)	0 (0.0%)	0.15
Relevant pericardial effusion	1 (0.8%)	0 (0.0%)	0.32	1 (1.3%)	0 (0.0%)	0.32

Composite primary endpoint: death, stroke, or bleeding BARC ≥ 3 after 30 days, attendance in the emergency department < 7 d related to the procedure and vascular complications. MACCE (major cerebral and cardiovascular events).

## Data Availability

The data supporting the reported results can be consulted in the archives of the cardiology unit of the Hospital.

## Abbreviations

AF	Atrial fibrillation
LAAO	Left atrial appendage occlusion
OP	Outpatient protocol
CP	Conventional protocol
